# Expression and prognostic impact of CD49b in human lung cancer

**DOI:** 10.1097/MD.0000000000028814

**Published:** 2022-02-11

**Authors:** Anna Tirilomi, Omar Elakad, Sha Yao, Yuchan Li, Marc Hinterthaner, Bernhard C. Danner, Philipp Ströbel, Theodor Tirilomis, Hanibal Bohnenberger, Alexander von Hammerstein-Equord

**Affiliations:** a Department of Cardio-Thoracic and Vascular Surgery, University Medical Center, Göttingen, Germany; b Institute of Pathology, University Medical Center, Göttingen, Germany.

**Keywords:** cluster of differentiation 49b, human lung cancer

## Abstract

Lung cancer remains the worldwide leading cause of cancer-related death. Currently, prognostic biomarkers for the detection and stratification of lung cancer are being investigated for clinical use. The surface protein cluster of differentiation 49b (CD49b) plays an important role in promoting cell proliferation and invasion in different tumor entities and blocking CD49b improved the tumor immune response. Overexpression of CD49b has been associated with unfavorable survival rates in several malignant tumor entities, such as prostate cancer, gastric cancer and colon cancer. Therefore, we aimed to analyze the protein expression of CD49b in patients with different types of lung cancer and additionally to identify the influence of CD49b on clinicopathological characteristics and overall survival.

Expression levels of CD49b were retrospective analyzed by immunohistochemistry in 92 cases of pulmonary adenocarcinoma (AC), 85 cases of squamous cell lung carcinoma (SQCLC) and 32 cases of small cell lung cancer (SCLC) and correlated with clinicopathological characteristics and patients’ overall survival.

A strong expression of CD49b was most seen in SQCLC (78%), followed by AC (48%) and SCLC (9%). All patients combined, strong expression of CD49b correlated significantly with poorer overall survival. However, an increased expression of CD49b correlated significantly with a poorer survival rate only in SQCLC. In AC and SCLC, no significant correlation could be demonstrated in this regard.

In our study, CD49b expression was associated with poor overall survival in patients with SQCLC. Accordingly, CD49b could serve as a new prognostic biomarker and, moreover, be a potential new drug target in SQCLC.

## 1 Introduction

Lung cancer is the leading cause of cancer-associated deaths worldwide and resulted in 1.8 million deaths in 2018.^[1,2]^ Lung cancer is projected to remain the leading cause of cancer-related death in the United States and in Germany in 2030.^[3,4]^ Based on histology, lung cancer is divided into nonsmall cell lung cancer (NSCLC) and small cell lung cancer (SCLC). Furthermore NSCLC accounts for more than 80% of lung cancer cases and can be subdivided into adenocarcinoma (AC, 40%) and squamous cell lung carcinoma (SQCLC, 25%–30%).^[5]^ The 5-year survival rate of lung cancer is about 20% being better for NSCLC than SCLC.^[6]^

Treatment of NSCLC is performed according to the extent of cancer (TNM-Classification) and stage (union for international cancer control-stage). If no contraindication, patients with stage I or II are treated with radical surgical resection.

Adjuvant chemotherapy in stage II and IIIA after surgical resection leads to the prevention of distant metastases after potential curative surgery.^[7]^ In case the tumor is not operable, radiotherapy, conventional or stereotactic, is considered. Currently, lung cancer is often detected only when curative surgery is no longer possible.^[8]^

To find the best adjuvant therapeutic procedure and to provide a prognostic estimate, molecular characterization can be used. So, lung cancer may be characterized into many molecularly heterogeneous subgroups, which should be treated individually.^[9,10]^ The surface protein cluster of differentiation 49b (CD49b) is a structural integrin alpha subunit (therefore also called Integrin alpha-2) with numerous functions and encoded by the CD49b gene in humans. It plays an important role in cell adhesion and cell surface-mediated signaling and is an extracellular matrix receptor for collagens and laminins.^[11]^ CD49b was abnormally overexpressed and associated with unfavorable survival rates in several malignant tumor entities, such as prostate cancer, gastric cancer and colon cancer.^[12–14]^

Recently, CD49b has been identified as a potential prognostic biomarker for cancer research. In liver carcinoma overexpression of CD49b might be a significant independent prognostic factor.^[15]^ In addition, in osteosarcoma CD49b expression was found to be associated with the presence of metastases and poor 5-year overall survival.^[16]^ In glioblastoma, CD49b was highly expressed and had a negative impact on patient survival.^[17]^

In the present study, we therefore analyzed the protein expression of CD49b in patients with different types of lung cancer and investigated the association of CD49b expression to clinicopathological characteristics and overall survival.

## 2 Methods

After identification of patients who underwent surgery for lung cancer in the Department of Thoracic and Cardiovascular Surgery of the Goettingen University Medical Center, tissue samples were processed regarding CD49b expression. Approval was obtained from the Ethics Committee of the University Medical Center Göttingen (#1-2-08). Informed consent was obtained from all patients. All procedures were conducted in accordance with the Declaration of Helsinki (October 2013 version) and institutional, state, and federal guidelines. Inclusion criteria for the study were adult patients (>18 years of age) and histology of AC, SQCLC or SCLC. Patients with histology other than AC, SQCLC or SCLC, and patients with unresectable tumor or those with neoadjuvant treatment were excluded from this study.

### 2.1 Immunohistochemical staining

First, tissue samples were assembled into tissue microarrays and then immunohistochemically stained as previously described.^[18]^

After incubation of the 2-mm tissue sections in EnVision Flex Target Retrieval Solution at low PH (Dako/agilent, Santa Clara CA), a 20-minute incubation with the primary antibody against CD49b (Sigma–Aldrich, St. Louis, MO 1:500) followed at room temperature. Polymeric secondary antibodies coupled to horseradish peroxidase (EnVision Flex+, Dako) and DAB (Dako) were applied for visualization. After counterstaining with Meyer hematoxylin tissue samples were analyzed by light microscopy considering staining intensity; negative = grade 0, weakly positive = grade 1, and strongly positive = grade 2.

### 2.2 Statistical analysis

Data collection was made using Microsoft Excel 2010 (Microsoft, Redmond, WA, USA). For statistical analysis GraphPad Prism (version 7.00 for Windows, GraphPad Software, La Jolla, CA, www.graphpad.com) was used. For analyzing correlation between clinicopathologic characteristics and CD49b protein expression Chi-square test and Student *t* test were used. Kaplan–Meier estimator was used to study patients’ survival in relation to CD49b expression and differences were calculated according to Mantel–Cox log-rank test. *P* values <.05 were considered statistically significant.

## 3 Results

### 3.1 Patient characteristics

In total 209 patients (140 males, 69 females) diagnosed with lung cancer were included in this study. The median age of patients was 67 years (range 39–85 years) and two-third of the patients were older than 60 years (141 patients, 67%). Most patients had suffered NSCLC; 92 patients of AC and 85 patients of squamous cell lung cancer. On the other side, 32 patients suffered from SCLC. In most patients, resection status was R0 (>90%). Detailed data regarding patients’ characteristics, clinical status, lymphatic metastasis, and degree of tumor differentiation are presented in Table 1.

**Table 1:** Patient characteristics.

Feature	Cases	AC	SQCLC	SCLC
Total	209	92	85	32
Age median (range)	67 (39-85)	67 (39-85)	67 (49-83)	66 (50-81)
Gender				
Male	140 (67%)	50 (36%)	67 (48%)	23 (16%)
Female	69 (33%)	42 (61%)	18 (26%)	9 (13%)
Age				
≤60	68 (33%)	29 (43%)	27 (40%)	12 (18%)
>60	141 (67%)	63 (45%)	58 (41%)	20 (14%)
Degree of differentiation				
I + II	133 (64%)	65 (49%)	68 (51%)	0 (0%)
III	76 (36%)	27 (36%)	17 (22%)	32 (42%)
Lymph node metastasis				
No	118 (59%)	56 (47%)	46 (39%)	16 (14%)
Yes	82 (41%)	35 (43%)	39 (48%)	8 (10%)
Clinical stage				
I + II	148 (74%)	69 (47%)	58 (39%)	21 (14%)
III + IV	51 (26%)	22 (43%)	26 (51%)	3 (6%)
Resection status				
R0	180 (91%)	82 (46%)	75 (42%)	23 (13%)
R1 + 2	18 (9%)	5 (28%)	9 (50%)	4 (22%)

AC = adenocarcinoma, SCLC = small cell lung cancer, SQCLC = squamous cell lung cancer.

### 3.2 Protein expression of CD49b in lung cancer patients and correlation with clinicopathological characteristics

**Figure 1:**
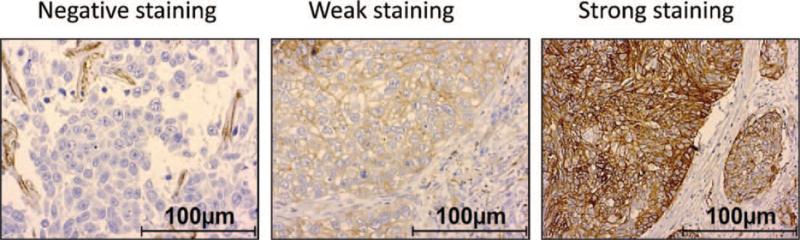
Representative immunohistochemical staining with negative (staining score 0), weakly positive (staining score 1) or strongly positive (staining score 2) immunostaining of CD49b in squamous cell lung cancer samples. Scale bar: 100 μm.

**Figure 2:**
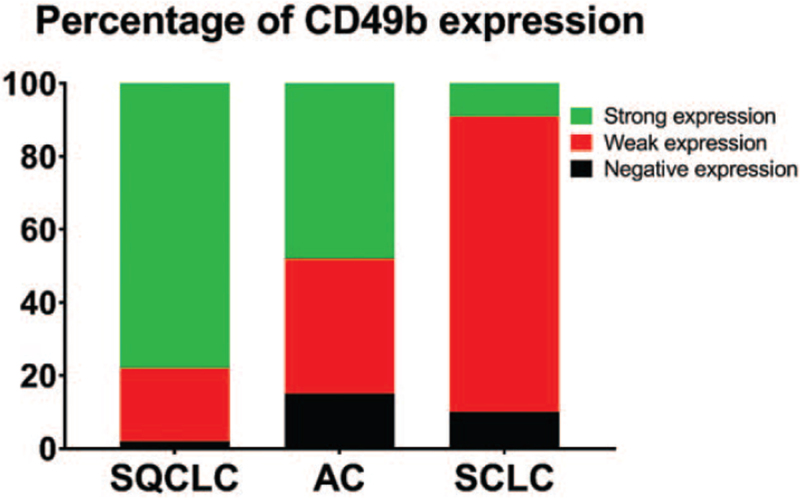
Protein expression of CD49b sorted by entity and categorized as negative, weakly positive, and strongly positive expression. AC = adenocarcinoma, SCLC = small cell lung cancer, SQCLC = squamous cell lung cancer.

Protein expression levels of CD49b in lung cancer tissue samples were analyzed by immunohistochemistry. According to staining intensity, expression of CD49b was described as negative (staining score 0), weakly positive (staining score 1) or strongly positive (staining score 2) and analyzed for each tumor entity (Fig. 1). A strongly positive immunostaining of CD49b was most seen in 66 patients (78%) in SQCLC, followed by 44 patients (48%) in AC and 3 patients (9%) in SCLC. CD49b was weakly positive expressed in 26 patients (81%) of SCLC, 34 patients (37%) in AC and 17 patients (20%) in SQCLC. A negative immunostaining of CD49b was most seen in 15 patients (15%) of AC, followed by 3 patients (10%) of SCLC and 2 patients (2%) of SQCLC (Fig. 2).

CD49b protein expression was significantly correlated with histological degree of differentiation in total cohort of patients (*P* = .0087) but not in individual subgroups. However, CD49b was found more often in clinical stages I + II (Table 2). Interestingly, in SCLC CD49b expression was more often increased in females than males (*P* = .0061). Within the subgroups, there were no statistically significant correlation between CD49b expression and any of the other clinicopathological features of patients (Tables 3–5).

**Table 2:** CD49b expression in adenocarcinoma, squamous cell lung cancer, and small cell lung carcinoma sorted by clinical features.

		CD49b		
Feature	Cases	−	+	*P*
Gender				
Male	140	58 (41%)	82 (59%)	.0627
Female	69	38 (55%)	31 (45%)	ns
Age				
≤60	68	35 (51%)	33 (49%)	.2646
>60	141	61 (43%)	80 (57%)	ns
Degree of differentiation				
I + II	133	52 (39%)	81 (61%)	.0087
III	76	44 (58%)	32 (42%)	^∗∗^
Lymph node metastasis				
No	118	55 (47%)	63 (53%)	.4713
Yes	82	34 (41%)	48 (59%)	ns
Clinical stage				
I + II	148	69 (47%)	79 (53%)	.359
III + IV	51	20 (39%)	31 (61%)	ns
Resection status				
R0	180	80 (44%)	100 (56%)	>.9999
R1 + 2	18	8 (44%)	10 (56%)	ns

*P* values are calculated according to Chi-square test. ns = not significant

∗∗ Highly significant.

**Table 3:** CD49b expression in squamous cell lung cancer samples sorted by clinical features.

		CD49b		
Feature	Cases	−	+	*P*
Gender				
Male	67	13 (19%)	54 (81%)	.2079
Female	18	6 (33%)	12 (67%)	ns
Age				
≤60	27	6 (22%)	21 (78%)	.9843
>60	58	13 (22%)	45 (78%)	ns
Degree of differentiation				
I + II	68	14 (21%)	54 (79%)	.4348
III	17	5 (29%)	12 (71%)	ns
Lymph node metastasis				
No	46	10 (22%)	36 (78%)	.8827
Yes	39	9 (23%)	30 (77%)	ns
Clinical stage				
I + II	58	12 (21%)	46 (79%)	.5278
III + IV	26	7 (27%)	19 (73%)	ns
Resection status				
R0	75	17 (23%)	58 (77%)	.976
R1 + 2	9	2 (22%)	7 (78%)	ns

*P* values are calculated according to Chi-square test.

**Table 4:** CD49b expression in adenocarcinoma samples sorted by clinical features.

		CD49b		
Feature	Cases	−	+	*P*
Gender				
Male	50	25 (50%)	25 (50%)	.6488
Female	42	23 (55%)	19 (45%)	ns
Age				
≤60	29	18 (62%)	11 (38%)	.1974
>60	63	30 (48%)	33 (52%)	ns
Degree of differentiation				
I + II	65	38 (58%)	27 (42%)	.061
III	27	10 (37%)	17 (63%)	ns
Lymph node metastasis				
No	56	30 (54%)	26 (46%)	.8421
Yes	35	18 (51%)	17 (49%)	ns
Clinical stage				
I + II	69	37 (54%)	32 (46%)	.7669
III + IV	22	11 (50%)	11 (50%)	ns
Resection status				
R0	82	42 (51%)	40 (49%)	.6262
R1 + 2	5	2 (40%)	3 (60%)	ns

*P* values are calculated according to Chi-square test.

**Table 5:** CD49b expression in small cell lung cancer samples sorted by clinical features.

		CD49b		
Feature	Cases	−	+	*P*
Gender				
Male	23	20 (87%)	3 (13%)	.0061
Female	69	38 (55%)	31 (45%)	^∗∗^
Age				
≤60	12	11 (92%)	1 (8%)	.8756
>60	20	18 (90%)	2 (10%)	ns
Degree of differentiation				
I + II	0	0 (0%)	0 (0%)	NA
III	32	29 (91%)	3 (9%)	
Lymph node metastasis				
No	16	15 (94%)	1 (6%)	.6015
Yes	8	7 (88%)	1 (13%)	ns
Clinical stage				
I + II	21	20 (90%)	1 (5%)	.094
III + IV	3	2 (67%)	1 (33%)	ns
Resection status				
R0	23	21 (91%)	2 (9%)	.5399
R1 + 2	4	4 (100%)	0 (0%)	ns

*P* values are calculated according to Chi-square test. NA = not applicable (because values are zero).

∗∗ Highly significant.

### 3.3 Impact of CD49b expression and survival

**Figure 3:**
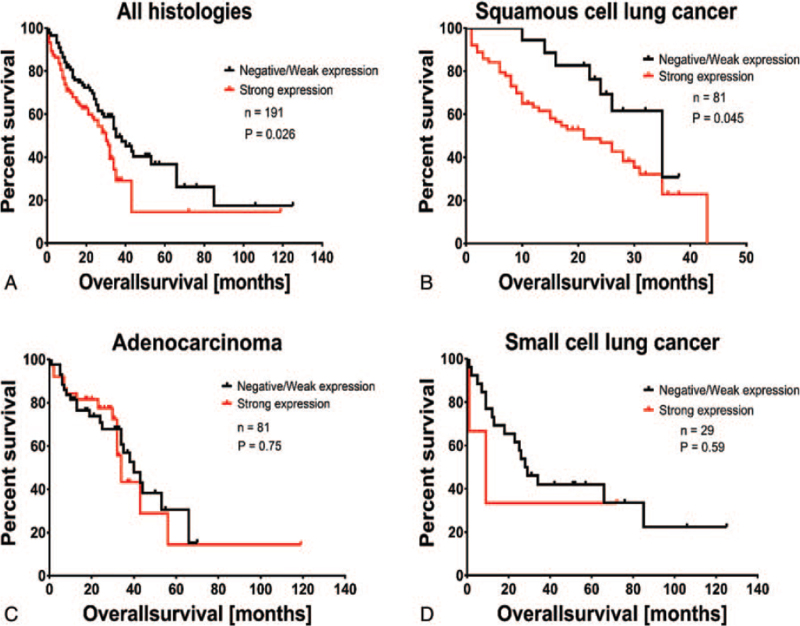
Kaplan–Meier analysis of OS in all patients (A), in patients with SQCLC (B), AC (C) and SCLC (D). The *P* value is from a log-rank test. AC = adenocarcinoma, OS = overall survival, SCLC = small cell lung cancer, SQCLC = squamous cell lung cancer.

Next, using Kaplan–Meyer-estimation, we examined the correlation between CD49b protein expression strength and overall survival in total samples combined and in each entity individually. The median follow-up time was 24 months (range, 1–125). In all entities combined, strong expression of CD49b correlated significantly with poorer overall survival (median survival 35 vs 30 months, hazard ratio = 0.6367, 95% confidence interval of ratio = 0.4271–0.9491 and *P* = .0284) (Fig. 3A**)**. Additionally, in patients with SQCLC strong expression of CD49b correlated significantly with poorer overall survival (median survival 35 vs 21 months, hazard ratio = 0.5128, 95% confidence interval of ratio = 0.2666–0.9863 and *P* = .0454) (Fig. 3B). The impact of CD49b on overall survival in patients with SQCLC was independent of the clinical features tested in Table 3. No significant correlation of CD49b expression with overall survival was found in patients of AC and SCLC (Fig. 3C and D).

## 4 Discussion

The survival rate for lung cancer is still strongly dependent on the stage at diagnosis. Most lung cancers are diagnosed when the cancer has already metastasized outside the lungs.^[19]^ Treatment of lung cancer is performed according to TNM-classification and union for international cancer control-stage. In early stages, patients are treated with radical surgical resection. From stage II and onwards, adjuvant therapy plays an important role.^[7]^

Finding prognostic biomarkers provides clues to the expected individual course of the disease so that treatment strategies can be adapted. The surface protein CD49b has been identified as a potential prognostic biomarker for cancer research in various entities.

Therefore, we investigated the protein expression of CD49b in patients with different types of lung cancer as well as the potential use of CD49b as a prognostic biomarker.

A strongly positive immunostaining of CD49b was most seen in SQCLC, followed by AC and SCLC. Interestingly, in SCLC CD49b expression was more often increased in females than males.

Regarding survival, a strong expression of CD49b correlated significantly with poorer overall survival in the entire group. More importantly, a correlation between poorer overall survival and CD49b expression was found in the SQCLC subgroup.

This leads to the consideration of whether therapy decisions should be intensified in the case of high levels of CD49b, which are associated with a poor prognosis. In perspective, it could be investigated whether CD49b could also be identified in the blood as a tumor marker and thus give an indication of the prognosis of the patients.

A correlation between poorer overall survival and CD49b expression has already been demonstrated in other cancer entities, such as osteosarcoma and glioblastoma. Moreover, CD49b could be identified as a prognostic biomarker with therapeutic consequence in these.^[16,17]^

In addition, it has already been demonstrated that CD49b plays an essential role in promoting cell proliferation and invasion in pancreatic cancer and blocking CD49b improved the tumor immune response by decreasing the phosphorylation level of signal transducer and activator of transcription 3-protein and suppressing programmed death ligand 1 expression in vivo.^[16]^ Therefore, CD49b may serve as a novel prognostic biomarker for solid cancer and a novel target for immune checkpoint blockade therapy.^[16]^ Specifically in SQCLC, CD49b could be evaluated as a potential prognostic biomarker that could also serve as a therapeutic target. The results of current study are limited due to single-center design. A multi-center study with larger patient cohort is suggested.

## Acknowledgments

We thank Jennifer Appelhans for her technical support.

## Author contributions

AH and HB conceived and supervised the project. AT, OE, SY, and YL executed experiments. AH, BCD, MH, TT, and PS contributed clinical samples and/or patient characteristics. AT, OE, TT, and AH wrote the manuscript with final approval of all authors.

**Conceptualization:** Omar Elakad, Sha Yao, Hanibal Bohnenberger, Alexander von Hammerstein-Equord.

**Data curation:** Omar Elakad, Sha Yao, Yuchan Li, Marc Hiterthaner, Bernhard C. Danner, Hanibal Bohnenberger.

**Formal analysis:** Omar Elakad, Yuchan Li, Hanibal Bohnenberger, Alexander von Hammerstein-Equord.

**Funding acquisition:** Bernhard C. Danner.

**Investigation:** Anna Tirilomi, Omar Elakad, Sha Yao, Yuchan Li, Philipp Ströbel, Hanibal Bohnenberger, Alexander von Hammerstein-Equord.

**Methodology:** Yuchan Li, Marc Hiterthaner, Bernhard C. Danner, Philipp Ströbel, Hanibal Bohnenberger.

**Project administration:** Philipp Ströbel, Hanibal Bohnenberger.

**Resources:** Philipp Ströbel.

**Software:** Yuchan Li.

**Supervision:** Philipp Ströbel, Theodor Tirilomis, Hanibal Bohnenberger, Alexander von Hammerstein-Equord.

**Validation:** Marc Hiterthaner, Theodor Tirilomis, Hanibal Bohnenberger.

**Visualization:** Hanibal Bohnenberger.

**Writing – original draft:** Anna Tirilomi.

**Writing – review & editing:** Theodor Tirilomis, Hanibal Bohnenberger, Alexander von Hammerstein-Equord.
